# Dual-Polarization radar rainfall prediction and rain gauge data

**DOI:** 10.1186/s13104-021-05693-7

**Published:** 2021-08-21

**Authors:** Tyson H. Walsh, Jesse W. Lansford, T. V. Hromadka, Prasada Rao

**Affiliations:** 1 Department of Mathematics, United States Military Academy, West Point, NY USA; 2 Department of Civil and Environmental Engineering, California State University, Fullerton, CA USA

**Keywords:** Hydrometeorology, Engineering, Floodplain management, Weather forecasting

## Abstract

**Objective:**

Reported rainfall data from multiple rain gauges and its corresponding estimate from Dual-Polarization (Dual-Pol) radar is presented here. The ordered set of data pairs were collected from multiple peer reviewed publications spanning across the last decade.

**Data description:**

Taken from multiple sources, the data set represents several radar sites and rain gauge sites combined for 12,734 data points. The data is relevant in various applications of hydrometeorology and engineering as well as weather forecasting. Further, the importance of accuracy in radar precipitation estimates continues to increase, necessitating the incorporation of as much data as possible.

## Objective

Prediction of rainfall using the upgrade to the Doppler Radar, the Dual-Polarization (Dual-Pol) technology relies on synthesizing the signal information that the radars receive back from the atmosphere. Processing this signal information yields a rainfall estimate relying on relationships between statistical regression equations and several other parameters. The upgrades to the technology in Dual-Pol radar came from advances made in 30 years of hardware and software research by the National Weather Service (agency of the United States government; www.weather.gov) and National Severe Storms Laboratory, located in Norman, Oklahoma (www.nssl.noaa.gov/). A Dual-Pol radar emits energy pulses along both the horizontal and vertical directions (with respect to the ground), unlike Doppler radar that emits energy only in horizontal direction. By differentiating between the horizontal and vertical signals, Dual-Pol estimates more specific information regarding the type of precipitation (i.e. snow, rain, hail), an aspect missing in Doppler radars. Researchers may use these rainfall estimates for study purposes by comparing the accuracy of the Dual-Pol radar estimated precipitation with the actual comparative rain gauge data. This data is relevant to other researchers involved in investigating the association of Dual-Pol radar data and rain gauge precipitation estimate relationships.

## Data description

Datasets generated and/or analyzed during the current study are available in the Figshare repository: https://doi.org/10.6084/m9.figshare.13103240 [1] (for review access prior to publication use this private link: https://figshare.com/s/4ed6abdce010ecf06dd9).

The data presented are radar data that have been pre-processed from published literature in the form of scatter plots and tables [3–12]. Specifically, these are “Dual-Pol radar derived rainfall estimates” coupled with the “observed local rain gauge values” collected from multiple storms and geographical domains, of which the majority are categorized via total storm accumulation. The data from the published graphs [3–12] was read using the plot digitizer (http://plotdigitizer.sourceforge.net/), which is a Java tool that digitizes data points from scanned plots. Note that the data used for analysis is *not* raw data; it is the end data obtained by the respective authors after applying standard corrections (if any) to the radar reflectivity and other parameters. The rain gauge data are also the final data as recorded by the gauges at the respective locations. The data comprise S and C-band radar types over 1 × 1 km areas with near-real time rainfall gauge collection utilizing high temporal resolution. There is no need for additional corrections or processing for the published data in the cited references. Assume that the displayed data points relating to the correlation between Dual-Pol radar readings versus measured precipitation, as shown in the various figures of the published papers shown in the references, are adequately processed and suitable for further analysis. Without the actual source data, the processed data supplied by the respective papers’ authors or digitizing the data directly from the papers used, populated the ordered pairs in the presented data set. Assume that standard procedures for processing the source data were used for the various papers assembled from the literature. These standard procedures as detailed by the respective authors relate to the geographical location of radar and gauge. Similarly, the rain gauge data are obtained from the papers’ authors or by digitizing the point data displayed in the respective paper’s diagrams and figures.

The data file (Table 1) comprises two columns of rainfall data; Gauge and Radar. Gauge consists of rainfall values in millimeters given by the recording rain gauge level following the precipitation event. Radar consists of estimated rainfall values in millimeters given by the Dual-Pol radar estimates prior to the precipitation event. Concatenating these two columns results in 12,734 ordered pairs.

**Table 1:** Overview of data files/data sets

Label	Name of data file/data set	File types (file extension)	Data repository and identifier (DOI or accession number)
Data file 1	DualPolWeatherData	Comma Separated Values (.csv)	https://doi.org/10.6084/m9.figshare.13103240 [2]

This data collection and publication effort does not include measurement site location or collection time. However, the data originate from referenced publications and therein contain such information.

## Limitations

The limitations in this study include the data of both the physical gauges and the Dual-Pol radar predictions. Although we assumed that the gauge data measurement to be the ground truth, factors like wind speed at ground level and the associated turbulence in the vicinity of gauge height can affect the accuracy of the measured readings. The radar beam is targeted towards the upper region of the precipitation cloud, and hence with distance from the radar, the error in Dual-Pol radar precipitation prediction increases. Given the vast region that the radar covers, choosing the appropriate gauge station can be a challenging task, and in regions where the gages are located densely. The limited number of volume scan sweeps and the attenuation might not be sufficient for intense moving storms. The quantitative precipitation estimation (QPE) algorithms in Dual-Pol radar have been maturing to include reliable estimation of attenuation, differential phase, and reflectivity. In this effort, we did not attempt to evaluate the QPE algorithm that the authors have used in their studies. While the number of studies that compared the Doppler radar predicted data with gauge data are many, the related studies for Dual-Pol radar are now gaining momentum and a limitation in this study can be the size of the data set that was used.

## Data Availability

Please see Table 1 and reference [2] for details and links to the data. The datasets generated during and/or analysed during the current study are available in the figshare repository: https://doi.org/10.6084/m9.figshare.13103240.

## Abbreviation

Dual-Pol: Dual-Polarization radar, referred to as Dual-Polarimetric radar as well
